# Association Between Weightbearing CT and MRI Findings in Progressive Collapsing Foot Deformity

**DOI:** 10.1177/10711007241231221

**Published:** 2024-02-28

**Authors:** Lynn Andres, Ricardo Donners, Dorothee Harder, Arne Burssens, Corina Nüesch, Nicola Krähenbühl

**Affiliations:** 1 Department of Orthopaedics, University Hospital Basel, Basel, Switzerland; 2 Department of Radiology and Nuclear Medicine, University Hospital Basel, Basel, Switzerland; 3 Department of Orthopaedics, University Hospital Ghent, Gent, Belgium

**Keywords:** imaging, weightbearing CT, deformity, progressive collapsing foot deformity

## Abstract

**Background:**

Weightbearing computed tomography (WBCT) scans allow for a better understanding of foot alignment in patients suffering from progressive collapsing foot deformity (PCFD). However, soft tissue integrity (eg, spring ligament complex or tibialis posterior tendon) cannot be easily assessed via WBCT. As performing both WBCT and magnetic resonance imaging (MRI) might not be cost effective, we aimed to assess whether there is an association between osseous and soft tissue findings in WBCT and MRI.

**Methods:**

In this observational study, a consecutive cohort of 24 patients of various stages of PCFD (mean age 51 ± 18 years) underwent WBCT scans and MRI. Twenty-four healthy individuals of similar age, body mass index (BMI), and sex with WBCT scans were used as a control group. In addition to of osseous sinus tarsi impingement, 4 commonly used 3-dimensional (3D) measurements (talocalcaneal overlap [TCO], talonavicular coverage [TNC], Meary angle [MA], axial/lateral) were obtained using a dedicated postprocessing software (DISIOR 2.1, Finland) on the WBCT data sets. Sinus tarsi obliteration, spring ligament complex, tibiospring ligament integrity, as well as tibialis posterior tendon degeneration were evaluated with MRI. Statistical analysis was performed for significant (*P* < .05) correlation between findings.

**Results:**

None of the assessed 3D measurements correlated with either spring ligament complex or tibiospring ligament tears. BMI and TCO were found to be associated with tibialis posterior tendon tears. Seventy-five percent of patients with osseous sinus tarsi impingement on WBCT also showed signs of sinus tarsi obliteration on MRI.

**Conclusion:**

Although WBCT reflects foot alignment and can reveal osseous sinus tarsi impingement in PCFD patients, the association between WBCT-based 3D measurements and ligament or tendon tears assessed via MRI is limited. WBCT appears complimentary to MRI regarding its diagnostic value. Both imaging options add important information and may impact decision making in the treatment of PCFD patients.

**Level of Evidence:**

Level IV, observational study.

## Introduction

Progressive collapsing foot deformity (PCFD) is a relatively common condition with progressive valgus hindfoot deformity and collapse of the longitudinal medial arch.^2,7,11^ Despite the fact that PCFD can be associated with posterior tibial tendon dysfunction and midfoot laxity, the etiology of this common foot deformity remains multifactorial.^7,20,24^ Studies have shown that progressive soft tissue degeneration, which typically increases over time, may impact the foot deformity.^1,3^ However, the association between specific ligament or tendon tears and foot deformity in PCFD has not yet been extensively studied. In addition, it is unclear whether age or body mass index (BMI) are associated with increased foot deformity or soft tissue degeneration assessed via weightbearing computed tomography (WBCT) or magnetic resonance imaging (MRI).

Traditionally, imaging in PCFD patients is done with weightbearing radiographs of the foot and ankle.^16,22^ However, common measurements used to evaluate foot alignment can be difficult to obtain without a margin of error.^13^ Although the accuracy of some parameters has been shown to be sufficient, factors such as the imaging quality as well as foot positioning during imaging affect measurements, resulting in poor repeatability.^13,16,22^ Conversely, 3-dimensional (3D) measurements derived from WBCT scans have been proven to be a more reliable alternative for deformity assessment.^12,13^ In recent studies, relevant WBCT-based measurements have been associated with peritalar joint subluxation and osseous sinus tarsi impingement.^2,4,11^ Such findings are especially of interest for clinicians as patients with significant osseous impingement may preferably be treated by realignment fusions rather than osteotomies.^12^

Because soft tissue integrity (eg, spring ligament complex or tibialis posterior tendon) cannot easily be assessed via WBCT, MRI helps obtain further information. This is especially important as soft tissue reconstruction is often required if surgery is performed. As performing both WBCT and MRI might not be cost effective, we aimed to assess whether there is an association between WBCT (alignment/osseous) and MRI (soft tissue) alterations. Additionally, we aimed to assess the correlation between WBCT/MRI findings and patient-specific factors such as age and BMI. A matched control group of healthy individuals was available for comparison. We hypothesized that (1) there is an association between specific WBCT-based 3D measurements and the presence of full or partial ligament and tendon tears on MRI; (2) osseous sinus tarsi impingement identified on WBCT is associated with sinus tarsi obliteration on MRI; and (3) age and BMI are associated with increased foot deformity, presence of osseous sinus tarsi impingement, and soft tissue degeneration.

## Materials and Methods

### Patients and Demographics

The Institutional Review Board (IRB) approved this observational study and informed consent was waived. The study included a consecutive cohort of 24 patients (mean age 51 ± 18 years) suffering from PCFD treated at the University Hospital of Basel between August 2021 and January 2023. Each patient underwent WBCT and MRI. Indication for imaging analysis was foot pain >6 months despite conservative treatment (physiotherapy and orthopaedic insoles). Diagnosis of PCFD was based on patients’ history, clinical examination, and weightbearing radiographs. Exclusion criteria were patients younger than 18 years and any prior foot surgery (on either side). In addition, patients with any history of foot trauma (on either side) were excluded. A control group of 24 healthy individuals with similar baseline characteristics (age, gender, side, BMI) was also included (WBCT, but no MRI available). Exclusion criteria for the control group were age <18 years, preexisting foot pain, history of any foot surgery (either side), or any foot trauma (either side). The healthy individuals were part of a larger cohort used in other studies.^12,13^ Baseline characteristics are shown in Table 1.

**Table 1.** Baseline Characteristics Assessed Using Simple Linear Models (Age, BMI) and Fisher Exact Tests (Sex, Side).

	Healthy	PCFD	*P* Values
Ankles, n	24	24	NA
Age, y, mean ± SD (range)	52±8 (40-66)	51±18 (19-81)	.835
Sex, female/male, n (%)	15 (63) / 9 (37)	12 (50) / 12 (50)	.561
Side, right/left, n (%)	11 (46) / 13 (54)	13 (54) / 11 (46)	.773
BMI, mean ± SD	25.3±3.4	27.5±5.0	.080

Abbreviations: BMI, body mass index; NA, not applicable; PCFD, progressive collapsing foot deformity.

### Imaging and Assessment

**Figure 1.**
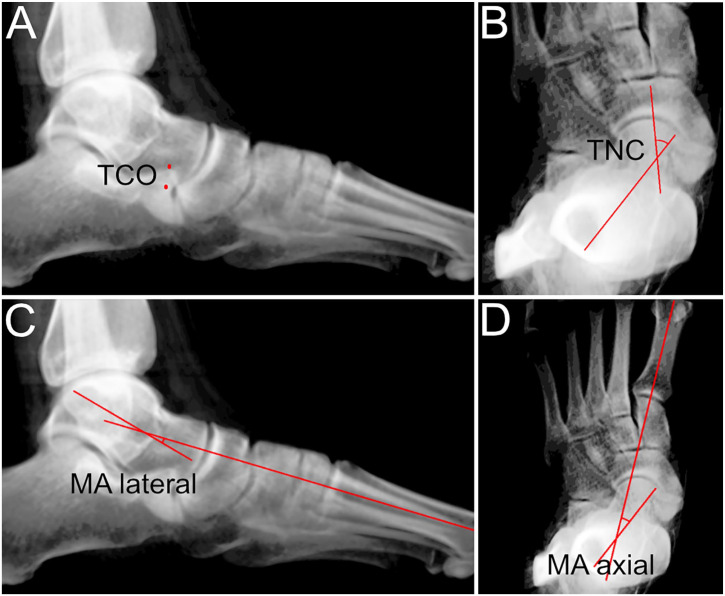
Semiautomated 3D measurement based on weightbearing computed tomography scans. (A) Lateral image of the foot showing the overlap between the calcaneal anterior process and the talus (TCO). (B) Dorsoplantar (DP) image of the foot showing talonavicular coverage (TNC) angle. (C) Lateral foot image showing lateral Meary angle (MA lateral). (D) DP image of the foot showing axial Meary angle (MA axial).

**Figure 2.**
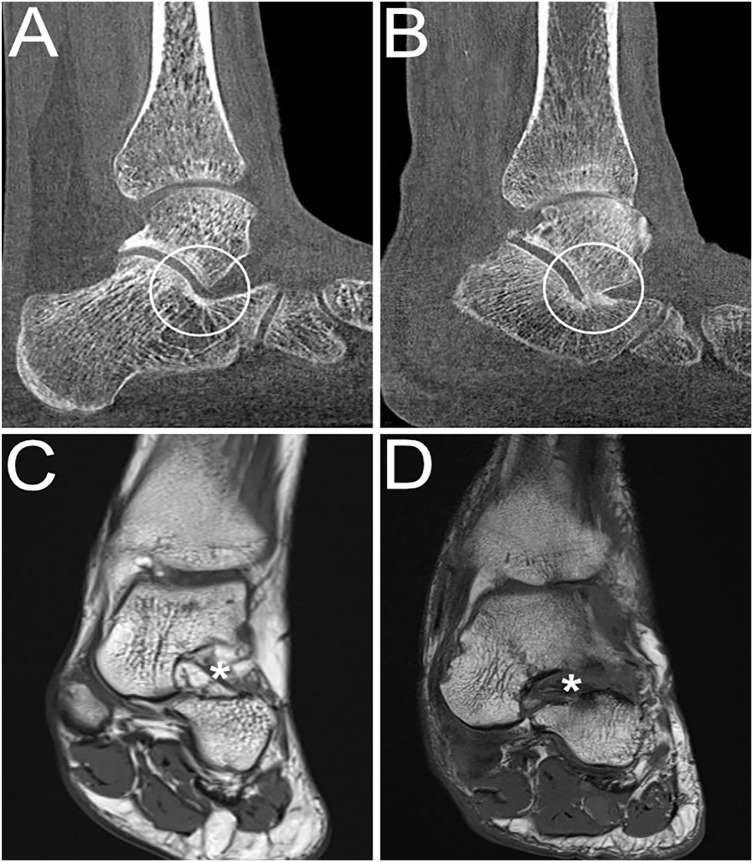
Weightbearing (WB) CT and MR images showing the sinus tarsi in PCFD patients. (A) Sinus tarsi without impingement and (B) sinus tarsi impingement visible on a sagittal WBCT image. (C) T1-weighted coronal MR image showing a normal sinus tarsi (*) and (D) T1-weighted coronal MR image showing complete sinus tarsi obliteration (*) with loss of physiological fat signal. CT, computed tomography; MR, magnetic resonance; PCFD, progressive collapsing foot deformity.

Every patient underwent WBCT imaging (Multitom Rax; Siemens Healthineers, Germany) in a neutral bipedal standing position. Healthy individuals underwent WBCT imaging in a neutral monopedal standing position (Planmed Verity; Planmed Oy, Finland). Alignment assessment was performed using a commercially available postprocessing software (DISIOR 2.1, Finland).^13^ In total, 4 clinically established 3D measurements were assessed (Figure 1): (1) talocalcaneal overlap (TCO, mm); (2) talonavicular coverage (TNC, degrees); (3) Meary angle lateral (MA lateral, degrees); and (4) Meary angle axial (MA axial, degrees). TCO and TNC were chosen because a previously published study has demonstrated that these 2 parameters correlate well with peritalar subluxation.^11^ MA lateral/axial were assessed because these 2 angles are frequently evaluated in PCFD patients with good correlation to deformity severity.^6,16,22^ Acceptable reliability was previously shown for the software segmentation/3D measurements.^12,13^ In addition to 3D measurements, osseous sinus tarsi impingement was assessed on WBCT images by a fellowship-trained subspecialized foot and ankle surgeon (N.K.).^12^ Osseous sinus tarsi impingement was defined according to a published protocol as immediate bony contact between talus and calcaneus at the level of the sinus tarsi (Figure 2).^12^

**Figure 3.**
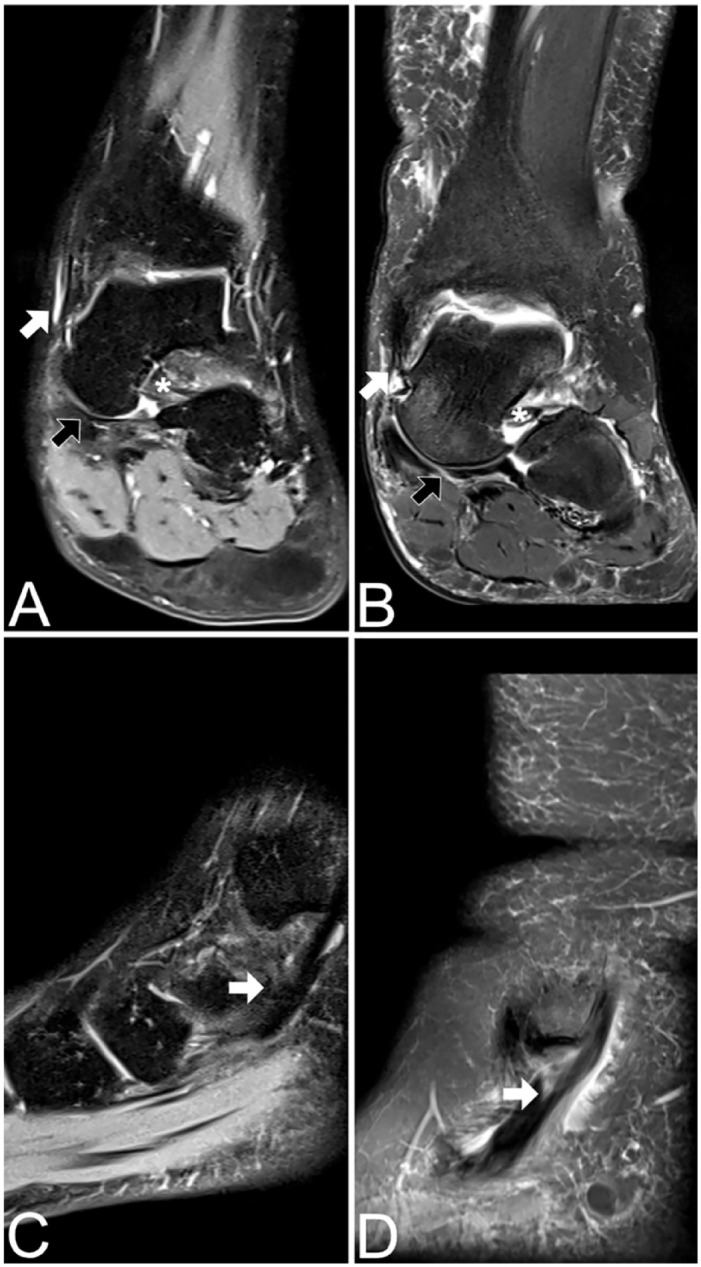
MR images showing tibiospring ligament, spring ligament, and tibialis posterior tendon in PCFD patients. (A) PD-weighted fat-saturated (FS) coronal image shows intact tibiospring (white arrow) and intact spring (black arrow) ligament. (B) PD-weighted FS coronal image shows complete tear with residual proximal fibers of the tibiospring ligament (white arrow) and a torn spring ligament (black arrow). (C) PD-weighted FS sagittal image shows intact tibialis posterior tendon. (D) PD-weighted FS sagittal image shows partial split-tear of the tibialis posterior tendon. PCFD, progressive collapsing foot deformity.

MRI is generally considered to be the imaging gold standard for evaluation of soft tissues around the hindfoot and allows for reliable assessment of the spring ligament complex, tibiospring ligament, tibialis posterior tendon, and of the sinus tarsi.^15,17-19,23^ MRI was performed in supine position on 3T machines in all patients (Magnetom Skyra; Siemens Healthineers). A standard clinical imaging protocol, including proton-density weighted coronal and sagittal, T2-weighted axial and T1-weighted axial and coronal sequences, was employed. A fellowship-trained subspecialized musculoskeletal radiologist (R.D.) assessed each MRI with special focus on the ligamentous and tendinous stabilizers of the plantar arch. The presence or absence of tears of the (1) spring ligament complex, (2) tibiospring ligament, and (3) tibialis posterior tendon as well as the presence or absence of (4) sinus tarsi obliteration was evaluated (Figures 2 and 3). The spring ligament complex, tibiospring ligament, and tibialis posterior tendon were graded as either intact (without partial- or full-thickness tears; group 1) or partially/fully torn (group 2), independent of where the tear was located. Sinus tarsi obliteration was defined as the loss of the physiological fat equivalent signal in the sinus tarsi and a lack of discernibility of the local interosseous and cervical ligaments.^10,15^

### Statistical Methods

All statistical analyses were performed in R (v4.1.2) using RStudio (v2022.12.0). Statistical significance level was *P* <.05. An a priori power analysis for regression was carried out before the study using the function *pwr.f2.test* from R-package *pwr*. With a power of 0.8, a significance level of .05, and 3 groups (healthy individual, PCFD patient with intact ligament, PCFD patient with partial or fully torn ligament) assessed, a sample size per group of 15 would be sufficient for a large effect size (Cohen *f²* above 0.4). Shapiro-Wilk tests were used to test the assumption of normality in our data. Differences in mean estimates between groups were assessed using simple linear regression. Compliance with model assumptions was checked using model residual plots. Differences in proportions between groups were assessed using Fisher exact tests. Probability for observing osseous sinus tarsi impingement was predicted using simple logistic regression fitted using all 48 subjects. The reported probabilities and CIs are the mean values over intervals of our response variables TCO (4.2-10.0, 10.1-15.0, and 15.1-25.5 mm), TNC (22.1-40, 40.1-50, and 50.1-64.3 degrees), and MA lateral (−3.4-20, 20.1-30, and 30.1-49.6 degrees), covering the ranges observed in our data.

## Results

### WBCT-Based Alignment and Soft Tissue Impairment

WBCT-based 3D measurements differed significantly between healthy individuals and PCFD patients, independent of spring ligament complex or tibiospring ligament integrity (Tables 2 and 3). BMI was significantly higher in PCFD patients with spring ligament complex tears compared to healthy individuals (Table 2). Although the assessed 3D measurements differed significantly between healthy individuals and PCFD patients with or without tibialis posterior tendon tears, only TCO differed significantly within PCFD subgroups (with or without tibialis posterior tendon tears; Table 4). Age and BMI differed significantly between healthy individuals and PCFD patients with tibialis posterior tendon tears and between the 2 subgroups of PCFD patients (Table 4).

**Table 2.** Group Comparison Using Simple Linear Models or Fisher Exact Test (STI).^a^

	Healthy (n = 24)	PCFD Spring Ligament Complex		*P* Values		
		Group 1 (n = 8)	Group 2 (n = 16)	Healthy vs Group 1	Healthy vs Group 2	Group 1 vs Group 2
Age, y, mean ± SD	52±8	45±23	54±18	.221	.620	.130
BMI, mean ± SD	25.3±3.4	25.9±4.5	28.4±5.2	.760	.031*	.177
STI, n (%)	0 (0)	3 (38)	9 (56)	NA	NA	.667
TCO, mm, mean ± SD	7.3±2.2	16.6±5.1	13.7±3.0	<.001*	<.001*	.034
TNC, degrees, mean ± SD	33.4±6.2	47.6±8.5	48.8±8.2	<.001*	<.001*	.705
MA lateral, degrees, mean ± SD	12.1±7.0	25.5±13.1	25.1±14.0	.004*	<.001*	.933
MA axial, degrees, mean ± SD	12.8±8.8	23.8±5.4	28.6±9.9	.004*	<.0001*	.211

Abbreviations: BMI, body mass index; MA, Meary angle; NA, not applicable; PCFD, progressive collapsing foot deformity; STI, sinus tarsi impingement; TCO, talocalcaneal overlap; TNC, talonavicular coverage.

a Group 1: intact; group 2: partially/fully torn.

* Statistically significant, *P* < .05.

**Table 3.** Group Comparison Using Simple Linear Models or Fisher Exact Test (STI).^a^

	Healthy (n = 24)	PCFD Tibiospring Ligament		*P* Values		
		Group 1 (n = 17)	Group 2 (n = 7)	Healthy vs Group 1	Healthy vs Group 2	Group 1 vs Group 2
Age, y, mean ± SD	52±8	50±19	55±16	.621	.683	.460
BMI, mean ± SD	25.3±3.4	27.2±4.6	28.3±6.1	.174	.113	.569
STI, n (%)	0 (0)	8 (47)	4 (57)	NA	NA	.999
TCO, mm, mean ± SD	7.3±2.2	14.4±4.1	15.2±4.0	<.001*	<.001*	.584
TNC, degrees, mean ± SD	33.4±6.2	47.6±7.6	50.3±9.8	<.001*	<.001*	.411
MA lateral, degrees, mean ± SD	12.1±7.0	22.5±12.0	31.7±15.3	.003*	<.001*	.054
MA axial, degrees, mean ± SD	12.8±8.8	25.9±8.6	29.7±9.4	<.001*	<.001*	.349

Abbreviations: MA, Meary angle; NA, not applicable; PCFD, progressive collapsing foot deformity; STI, sinus tarsi impingement; TCO, talocalcaneal overlap; TNC, talonavicular coverage.

a Group 1: intact; group 2: partially/fully torn.

* Statistically significant, *P* < .05.

**Table 4.** Group Comparison Using Simple Linear Models or Fisher Exact Test (STI).^a^

	Healthy (n = 24)	PCFD Tibialis Posterior Tendon		*P* Values		
		Group 1 (n = 20)	Group 2 (n = 4)	Healthy vs Group 1	Healthy vs Group 2	Group 1 vs Group 2
Age, y, mean ± SD	52±8	48±17	68±13	.302	.032*	.008*
BMI, mean ± SD	25.3±3.4	26.6±4.1	32.1±7.2	.296	.004*	.018*
STI, n (%)	0 (0)	8 (40)	4 (100)	NA	NA	.093
TCO, mm, mean ± SD	7.3±2.2	13.9±3.4	18.7±4.7	<.001*	<.001*	.004*
TNC, degrees, mean ± SD	33.4±6.2	47.8±8.0	51.3±9.3	<.001*	<.001*	.385
MA lateral, degrees, mean ± SD	12.1±7.0	23.9±13.2	31.5±14.7	<.001*	.002*	.198
MA axial, degrees, mean ± SD	12.8±8.8	26.6±8.7	29.4±10.2	<.001*	.001*	.565

Abbreviations: BMI, body mass index; MA, Meary angle; NA, not applicable; PCFD, progressive collapsing foot deformity; STI, sinus tarsi impingement; TCO, talocalcaneal overlap; TNC, talonavicular coverage.

a Group 1: intact; group 2: partially/fully torn.

* Statistically significant, *P* < .05.

### Osseous Sinus Tarsi Impingement and Sinus Tarsi Obliteration

The percentage of PCFD patients with evidence of osseous sinus tarsi impingement on WBCT did not change significantly whether ligament or tendon tears on MRI were present (Tables 2-4). Patients with osseous sinus tarsi impingent on WBCT (n = 12) also showed signs of sinus tarsi obliteration on MRI in 75% (n = 9) of cases. By comparison, patients without osseous sinus tarsi impingement on WBCT (n = 12) showed signs of sinus tarsi obliteration on MRI in 42% (n = 5) of cases. Although the baseline characteristics and imaging parameters did not differ between patients with either osseous sinus tarsi impingement or sinus tarsi obliteration, WBCT-based 3D measurements, with the exception of MA axial, differed between PCFD patients with or without sinus tarsi impingement (Table 5). The probability of predicting osseous sinus tarsi impingement was >0.8 if TCO was ≥15 mm, TNC was ≥50 degrees, or MA lateral was ≥30 degrees (Table 6).

**Table 5.** Group Comparison Using Simple Linear Models or Fisher Exact Test.

	Sinus Tarsi Impingement		*P* Value	Sinus Tarsi Obliteration		*P* Value
	No (n = 12)	Yes (n = 12)		No (n = 10)	Yes (n = 14)	
Age, y, mean ± SD	44±18	58±15	.054	42±20	58±13	.024*
BMI, mean ± SD	25.8±4.2	29.3±5.2	.082	25.0±4.4	29.3±4.7	.035*
Spring ligament complex^a^, n (%)	7 (58)	9 (75)	.667	6 (60)	10 (71)	.673
Tibiospring ligament^a^, n (%)	3 (25)	4 (33)	>.999	4 (40)	3 (21)	.393
Tibialis posterior tendon^a^, n (%)	0 (0)	4 (33)	.093	0 (0)	4 (29)	.114
TCO, mm, mean ± SD	12.3±2.5	17.1±3.7	.001*	13.6±4.4	15.4±3.6	.261
TNC, degrees, mean ± SD	42.7±4.1	54.2±7.0	<.001*	46.5±7.5	49.8±8.6	.350
MA lateral, degrees, mean ± SD	18.2±11.7	32.1±11.6	.008*	27.0±13.1	23.9±14.0	.585
MA axial, degrees, mean ± SD	23.9±9.2	30.2±7.5	.08	23.7±7.5	29.4±9.1	.119

Abbreviations: BMI, body mass index; MA, Meary angle; TCO, talocalcaneal overlap; TNC, talonavicular coverage.

a Partially or fully torn.

* Statistically significant, *P* < .05.

**Table 6.** Probability of Predicting Sinus Tarsi Impingement.

TCO, mm	
≤10	<.001 (<.001-.22)
10-15	.23 (.09-.55)
≥15	.95 (.70-.99)
TNC, degrees	
≤40	<.001 (<.001-.28)
40-50	.13 (.32-.64)
≥50	.96 (.52-.99)
MA lateral, degrees	
≤20	.16 (.04-.51)
20-30	.49 (.27-.72)
≥30	.81 (.47-.94)

Abbreviations: MA, Meary angle; TCO, talocalcaneal overlap; TNC, talonavicular coverage.

## Discussion

A retrospective analysis of PCFD patients was performed to assess whether there is an association between WBCT-based measurements, MRI findings, and patient-specific characteristics. The 3 most important findings are as follows: (1) foot deformity (assessed via TCO), advanced age, and higher BMI are associated with tibialis posterior tendon tears; (2) osseous sinus tarsi impingement diagnosed on WBCT is not associated with ligament or tendon tears on MRI; and (3) MRI overestimates the presence of osseous sinus tarsi impingement (identified on WBCT) in approximately 42% in our population.

In recent years, WBCT has become increasingly popular in the evaluation of PCFD patients.^2,4,5,8,9,11-14^ Several studies have indicated that the assessment of the peritalar bones and joints under weightbearing conditions has major advantages compared to imaging options in supine position.^2,4,8,12,13^ Notably, an association between osseous sinus tarsi impingement and several WBCT-based 3D parameters (TCO, TNC, MA lateral) was evident in the present study. This finding is in accordance with earlier reports.^9,12^ It is important to mention that a TCO ≥15 mm or a TNC ≥50 degrees increased the probability of predicting osseous sinus tarsi impingement >0.9 in our cohort. Interestingly, a recent study showed a strong association between TCO and peritalar subluxation.^11^ This may underline the utility of this specific measurement in the assessment of PCFD patients. Several studies have additionally shown an acceptable reliability of TCO assessment on weightbearing radiographs (interobserver reliability >0.9 and intraobserver reliability >0.8) as well as an association between TCO and foot rigidity.^12,13^ This is especially of interest as weightbearing radiographs are still widely used in imaging of PCFD patients, with foot flexibility as an important factor in PCFD classification.^21^

Although none of the assessed WBCT-based findings were associated with spring ligament complex or tibiospring ligament tears, a greater TCO was evident in the presence of tibialis posterior tendon tears. Of note, a previous report indicated an association between osseous sinus tarsi impingement (identified on WBCT) and tibialis posterior tendon degeneration.^3^ This finding could not be confirmed in the present study. A possible explanation might be that different definitions of sinus tarsi impingement assessed on WBCT are available in literature.^3,5,9,12,14^ Although some studies defined osseous sinus tarsi impingement as direct bony contact, others include indirect signs such as cyst formation or defined a minimum distance between the talus and calcaneus at the sinus tarsi region.^3,5,9,12,14^ Interestingly, the presence of sinus tarsi obliteration on MRI overestimated the presence of osseous sinus tarsi impingement as defined on WBCT in approximately 42% in our population. Although there are no comparable data available in the literature, it seems apparent that changes in peritalar joint coverage, which appear under load application, impact the visibility of signs of osseous sinus tarsi impingement on WBCT.^11^ This is especially of interest for clinicians as patients with significant osseous impingement may preferably be treated with realignment fusions rather than osteotomies.^12^

Tibialis posterior tendon tears were associated with an increased BMI and advanced age in the present study. The relationship between BMI or age and foot deformity or soft tissue impairment in PCFD patients is controversially discussed in literature.^7,12^ Previous literature on this topic is limited. Greisberg et al^7^ did not find a correlation between age and foot deformity. However, standard radiographs and non-WBCT scans were used in his study.^7^ A later WBCT-based study found that osseous sinus tarsi impingement as well as advanced foot rigidity is more frequently seen in comparably older patients.^12^ Unfortunately, BMI was not considered in either study. Nevertheless, our study indicates that an increased BMI may impact structural changes in PCFD patients. If applicable, weight reduction should therefore be recommended as part of the conservative treatment before surgical options are discussed. This is especially true for overweight patients of comparably younger age.

This study has several limitations. First, MRI interpretations were simplified and limited to the presence or absence of a tendon or ligament tear. This was done to improve repeatability of our findings and facilitate the analyses. We appreciate that degenerative tissue changes without tears, such as thickening or signal alteration, may show improved correlation with WBCT measurements. Second, we only looked at each ligament and tendon individually. A combined analysis of these structures may also be of interest. Third, the usage of a commercially available software to evaluate 3D measurements needs to be critically evaluated as it remains difficult to understand the full process behind the assessment; therefore, inaccurate measurements may be missed in data analysis. Fourth, weightbearing radiographs are still widely used in the initial assessment of PCFD patients because advanced imaging options are not always available or necessary. Fifth, the total number of patients included in this study was rather small; therefore, the used sample size was too low to be able to detect medium differences. Consequently, statistical comparison (especially of subgroups) has to be interpreted with care.

To conclude, the relationship between WBCT-based 3D measurements and soft tissue impairment assessed via MRI is limited. Perhaps not surprisingly, we found partial or complete rupture of the tibialis posterior tendon more likely to occur in comparably older and overweight PCFD patients with an increased TCO. WBCT may not replace MRI and MRI does not replace WBCT regarding the diagnostic value of each. Both imaging options add different but important information possibly impacting decision making in the treatment of PCFD patients.

## Supplemental Material

sj-pdf-1-fai-10.1177_10711007241231221 – Supplemental material for Association Between Weightbearing CT and MRI Findings in Progressive Collapsing Foot Deformity Supplemental material, sj-pdf-1-fai-10.1177_10711007241231221 for Association Between Weightbearing CT and MRI Findings in Progressive Collapsing Foot Deformity by Lynn Andres, Ricardo Donners, Dorothee Harder, Arne Burssens, Corina Nüesch and Nicola Krähenbühl in Foot & Ankle International

## References

[1] Brodell JD Jr MacDonald A Perkins JA Deland JT Oh I. Deltoid-spring ligament reconstruction in adult acquired flatfoot deformity with medial peritalar instability. Foot Ankle Int. 2019;40(7):753-761. doi:10.1177/1071100719839176 30902021

[2] de Cesar Netto C Godoy-Santos AL Saito GH , et al Subluxation of the middle facet of the subtalar joint as a marker of peritalar subluxation in adult acquired flatfoot deformity: a case-control study. J Bone Joint Surg Am. 2019;101(20):1838-1844. doi:10.2106/JBJS.19.00073 31626008

[3] de Cesar Netto C Saito GH Roney A , et al Combined weightbearing CT and MRI assessment of flexible progressive collapsing foot deformity. Foot Ankle Surg. 2021;27(8):884-891. doi:10.1016/j.fas.2020.12.003 33358266

[4] de Cesar Netto C Silva T Li S , et al Assessment of posterior and middle facet subluxation of the subtalar joint in progressive flatfoot deformity. Foot Ankle Int. 2020;41(10):1190-1197. doi:10.1177/1071100720936603 32590925

[5] Dibbern KN Li S Vivtcharenko V , et al Three-dimensional distance and coverage maps in the assessment of peritalar subluxation in progressive collapsing foot deformity. Foot Ankle Int. 2021;42(6):757-767. doi:10.1177/1071100720983227 33504217

[6] Ellis SJ Deyer T Williams BR , et al Assessment of lateral hindfoot pain in acquired flatfoot deformity using weightbearing multiplanar imaging. Foot Ankle Int. 2010;31(5):361-371. doi:10.3113/FAI.2010.0361 20460061

[7] Greisberg J Hansen ST Jr Sangeorzan B. Deformity and degeneration in the hindfoot and midfoot joints of the adult acquired flatfoot. Foot Ankle Int. 2003;24(7):530-534. doi:10.1177/107110070302400704 12921357

[8] Jeng CL Rutherford T Hull MG Cerrato RA Campbell JT. Assessment of bony subfibular impingement in flatfoot patients using weight-bearing CT scans. Foot Ankle Int. 2019;40(2):152-158. doi:10.1177/1071100718804510 30293451

[9] Kim J Rajan L Fuller R , et al Radiographic cutoff values for predicting lateral bony impingement in progressive collapsing foot deformity. Foot Ankle Int. 2022;43(9):1219-1226. doi:10.1177/10711007221099010 35699393

[10] Klein MA Spreitzer AM. MR imaging of the tarsal sinus and canal: normal anatomy, pathologic findings, and features of the sinus tarsi syndrome. Radiology. 1993;186(1):233-240. doi:10.1148/radiology.186.1.8416571 8416571

[11] Knutson K Peterson AC Lisonbee RJ Hintermann B Krähenbühl N Lenz AL. Joint coverage analysis in progressive collapsing foot deformity. J Orthop Res. 2023;41(9):1965-1973. doi:10.1002/jor.25543 36891918 PMC10491741

[12] Krahenbuhl N Kvarda P Susdorf R , et al Assessment of progressive collapsing foot deformity using semiautomated 3D measurements derived from weightbearing CT scans. Foot Ankle Int. 2022;43(3):363-370. doi:10.1177/10711007211049754 34617817

[13] Kvarda P Krahenbuhl N Susdorf R , et al High reliability for semiautomated 3D measurements based on weightbearing CT scans. Foot Ankle Int. 2022;43(1):91-95. doi:10.1177/10711007211034522 34353147

[14] Lalevee M Barbachan Mansur NS Rojas EO , et al Prevalence and pattern of lateral impingements in the progressive collapsing foot deformity. Arch Orthop Trauma Surg. 2023;143(1):161-168. doi:10.1007/s00402-021-04015-7 34213577

[15] Lee KB Bai LB Park JG Song EK Lee JJ. Efficacy of MRI versus arthroscopy for evaluation of sinus tarsi syndrome. Foot Ankle Int. 2008;29(11):1111-1116. doi:10.3113/FAI.2008.1111 19026205

[16] Lee KM Chung CY Park MS Lee SH Cho JH Choi IH. Reliability and validity of radiographic measurements in hindfoot varus and valgus. J Bone Joint Surg Am. 2010;92(13):2319-2327. doi:10.2106/JBJS.I.01150 20926727

[17] Lektrakul N Chung CB Lai Y , et al Tarsal sinus: arthrographic, MR imaging, MR arthrographic, and pathologic findings in cadavers and retrospective study data in patients with sinus tarsi syndrome. Radiology. 2001;219(3):802-810. doi:10.1148/radiology.219.3.r01jn31802 11376274

[18] Mengiardi B Pinto C Zanetti M. Spring ligament complex and posterior tibial tendon: MR anatomy and findings in acquired adult flatfoot deformity. Semin Musculoskelet Radiol. 2016;20(1):104-115. doi:10.1055/s-0036-1580616 27077591

[19] Mengiardi B Zanetti M Schottle PB , et al Spring ligament complex: MR imaging-anatomic correlation and findings in asymptomatic subjects. Radiology. 2005;237(1):242-249. doi:10.1148/radiol.2371041065 16118154

[20] Myerson MS Badekas A Schon LC. Treatment of stage II posterior tibial tendon deficiency with flexor digitorum longus tendon transfer and calcaneal osteotomy. Foot Ankle Int. 2004;25(7):445-450. doi:10.1177/107110070402500701 15319100

[21] Myerson MS Thordarson DB Johnson JE , et al Classification and nomenclature: progressive collapsing foot deformity. Foot Ankle Int. 2020;41(10):1271-1276. doi:10.1177/1071100720950722 32856474

[22] Sangeorzan BJ Mosca V Hansen ST Jr. Effect of calcaneal lengthening on relationships among the hindfoot, midfoot, and forefoot. Foot Ankle. 1993;14(3):136-141. doi:10.1177/107110079301400305 8491427

[23] Toye LR Helms CA Hoffman BD Easley M Nunley JA. MRI of spring ligament tears. AJR Am J Roentgenol. 2005;184(5):1475-1480. doi:10.2214/ajr.184.5.01841475 15855099

[24] Zanolli DH Glisson RR Nunley JA 2nd Easley ME. Biomechanical assessment of flexible flatfoot correction: comparison of techniques in a cadaver model. J Bone Joint Surg Am. 2014;96(6):e45. doi:10.2106/JBJS.L.00258 24647512

